# Molecular characterization and phylogenetic analysis of porcine epidemic diarrhea virus strains circulating in China from 2020 to 2021

**DOI:** 10.1186/s12917-022-03481-4

**Published:** 2022-11-08

**Authors:** Hong Zhuang, Leilei Sun, Xiaobo Wang, Min Xiao, Long Zeng, Haoran Wang, Hongfu Yang, Feng Lin, Chuang Wang, Liting Qin, Chengbao Wang

**Affiliations:** 1 College of Veterinary Medicine, Northwest A&F University, Yangling, 712100 China; 2 Shandong New Hope Liuhe Group Co., Ltd., Qingdao, 266000 China; 3 Qingdao Jiazhi Biotechnology Co., Ltd., Qingdao, 266000 China; 4 Zhengbang Group Co., Ltd., Nanchang, 330000 China

**Keywords:** PEDV, Spike gene, Phylogenetic analysis, S-INDEL like strain, Recombination

## Abstract

**Background:**

Porcine epidemic diarrhea virus (PEDV), an enteric coronavirus, has become the major causative agent of acute gastroenteritis in piglets since 2010 in China.

**Results:**

In the current study, 91 complete spike (S) gene sequences were obtained from PEDV positive samples collected from 17 provinces in China from March 2020 to March 2021. A phylogenetic analysis showed that 92.3% (84 out of 91) of the identified strains belonged to GII subtype, while 7.7% (7 out of 91) were categorized as S-INDEL like strains and grouped within GI-c clade. Based on a recombination analysis, six of S-INDEL like strains were recombinant strains originated from S-INDEL strain FR/001/2014 and virulent strain AJ1102. In addition, PEDV variant strains (CH/GDMM/202012, CH/GXDX/202010 et al) carrying novel insertions (360QGRKS364 and 1278VDVF1281) in the S protein were observed. Furthermore, the deduced amino acid sequences for the S protein showed that multiple amino acid substitutions in the antigenic epitopes in comparison with the vaccine strains.

**Conclusions:**

In conclusion, these data provide novel molecular evidence on the epidemiology and molecular diversity of PEDV in 2020–2021. This information may help design a strategy for controlling and preventing the prevalence of PEDV variant strains in China.

**Supplementary Information:**

The online version contains supplementary material available at 10.1186/s12917-022-03481-4.

## Background

Coronaviruses (CoVs) can infect a wide variety of animals, and cause respiratory, enteric, and other diseases [1]. So far, the most relevant enteric coronaviruses in pigs include porcine epidemic diarrhea virus (PEDV), transmissible gastroenteritis virus (TGEV), and recently identified viruses, such as porcine deltacoronavirus (PDCoV) and swine acute diarrhea syndrome coronavirus (SADS-CoV) [2, 3]. Among these CoVs, PEDV has the highest detection rate and up to 100% mortality in neonatal piglets. The acute diarrhea caused by PEDV was characterized by severe vomiting, dehydration, and watery diarrhea [4]. Outbreaks of PED brought huge economic losses to pig production worldwide [5].

PEDV is an enveloped virus, whose genome is composed of a positive sense, single-stranded, and non-segmented RNA with a size of approximately 28 kb. PEDV genome encodes open reading frame (ORF) 1ab, spike (S), ORF3, envelope (E), membrane (M), and nucleoprotein (N) from 5′ to 3′ untranslated region (UTR) [6]. S protein is a glycoprotein peplomer located on the viral surface, and contains 1383–1386 amino acids (aa) in most strains. Despite the S protein of PEDV cannot be demarcated by a protease cleavage site, it is divided into S1 and S2 subunits based on the homology with other coronaviruses [7, 8]. The receptor for PEDV is still unknown. However, S1 protein has been shown to bind to sialic acid glycans and the porcine aminopeptidase N (pAPN) is a receptor binding domain for facilitating viral invasion. S2 subunit is responsible for membrane fusion [9, 10]. S protein is also the main target for inducing neutralizing antibodies. The neutralizing epitope region COE (499–638 aa), four neutralizing B cell epitopes S1^A^ (435–485 aa), SS2 (748–755 aa), SS6 (764–771 aa), and 2C10 (1368–1374 aa) have been identified on the S protein [11–14]. In addition, a specific linear B-cell epitope SE16 (722-731aa) had been identified to be required for a reactivity with the mAb 2E10. The epitope SE16 was localized on the surface of PEDV S protein [15].

PEDV was first reported in the United Kingdom in 1971 [16]. In China, PEDV was first identified and isolated in 1984. Since then, PEDV infection occurred sporadically and regionally. In 2010, a high virulent strain of PEDV emerged on pig farms in southern China, and caused up to 100% mortality in newborn piglets, and immediately swept throughout the country [17]. The presence of variant strain in China was identified by the detection of a field CH/FJND-3/2011 strain [18]. The AJ1102 strain isolated from a PEDV positive farm had become the prevalent variant for the time [19].

The epidemiological survey proceeded from February 2011 to March 2014 in 29 provinces of China showed that the PEDV positive rates for samples and pig farms were 61.10–78.49% and 71.43–83.47%, respectively. Genetic drift could be confirmed mainly by the genetic variation of S protein as compared with the Chinese commercialized vaccine strain CV777 [20, 21]. PEDV is mainly classified into two genotypes GI (classical) and GII (variant) on the basis of *S* gene [18]. In 2013, new PEDV variants containing new insertion and deletion in *S* gene versus prototype strain were reported in USA [22]. Subsequently, these variants, named S-INDEL-variant, were also detected and isolated in China [4]. S-INDEL strains were associated with milder clinical signs and lower mortality in suckling pigs.

Currently, PEDV is still the main pathogen that leads to the death of piglets on pig farms in China. High morbidity, variation, and recombination of viral genomes make it hard to prevent and control the prevalence of PEDV. To better understand the prevalence and molecular characteristics of PEDV in different regions of China, 91 PEDV positive samples were collected from 17 provinces of China. The full-length *S* genes were sequenced and analyzed with a focus on the variation of the neutralizing epitopes, the emergence of S-INDEL strains, and potential recombinant between different strains. These data systematically described the genetic and evolutionary characteristics of PEDV field strains in China from 2020 to 2021, and might promote the development of novel effective vaccines.

## Results

### Prevalence analysis and genome characteristics of PEDV based on S gene in China

In the PEDV positive farms, 115 clinical samples were found to be positive for PEDV based on the RT-PCR detection (Table 1). Among them, the *S* genes of 91 PEDV positive samples obtained in 17 provinces in China from 2020 to 2021 were successfully amplified and sequenced. This epidemiological survey covered the major pig-raising provinces in China (Table 2).

**Table 1:** Detection rates of PEDV from Mar 2020 to Mar 2021 in China

Month	2020			2021	Sum
	Mar ~ Jun	Jul ~ Sep	Oct ~ Dec	Jan ~ Mar	
Total samples	260	130	203	88	681
Samples sequenced	21	14	46	10	91
Positive samples	28	21	51	15	115
Positive rate (%)	10.8	16.2	25.1	17.0	16.9

**Table 2:** PEDV distribution in 17 provinces in China from March 2020 to March 2021 (The abbreviation of sampling provinces were given in supplementary Table 1)

PEDV negative province	PEDV positive province reported	PEDV positive province in this study
Tibet	Xinjiang, Qinghai, Gansu, Ningxia, Chongqing, Shanxi, Zhejiang, Beijing, Tianjin, Heilongjiang, Jilin, Hainan, Taiwan, Shanghai	Inner Mongoria, Liaoning, Hebei, Shandong, Shaanxi, **Henan**, Anhui, **Jiangsu**, **Sichuan**, Hubei, Guizhou, Yunnan, Guangxi, Hunan, **Jiangxi**, Fujian, Guangdong

Note: Bold letters stand for case of S-INDEL like strain

**Fig. 1:**
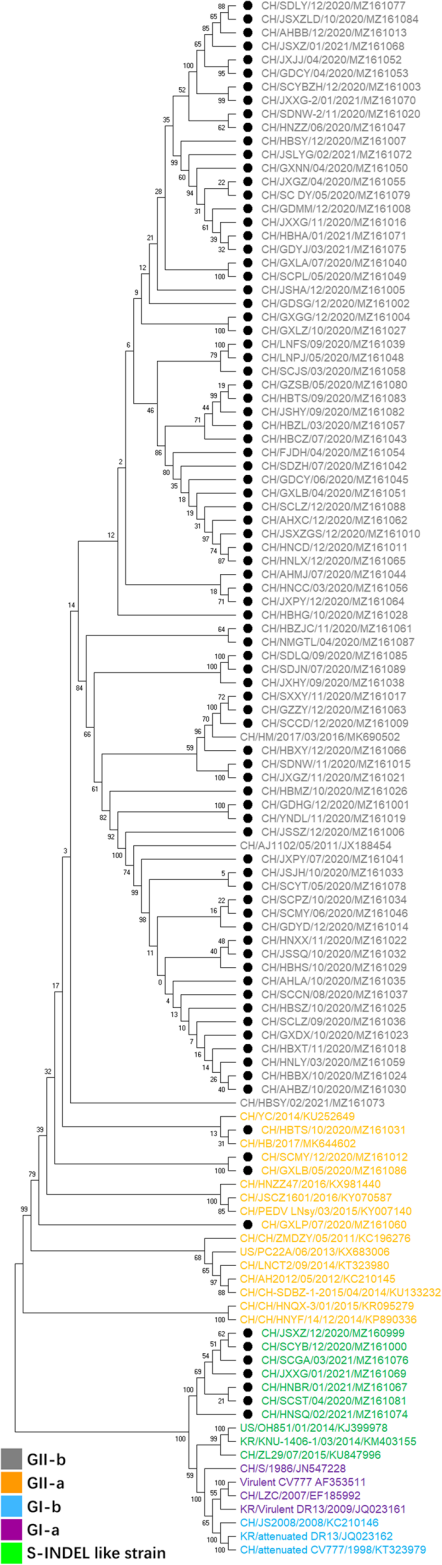
Geographical distribution and phylogenetic analysis of PEDV positive samples in China. Phylogenetic analysis of PEDV strains based on 91 *S* gene sequences identified in this study and 24 reference *S* gene sequences in Genbank. Multiple nucleotide sequence alignments are performed with ClustalW algorithm using MEGA 6.0 software. A neighbor-joining method based phylogenetic tree is automatically constructed with 1000 bootstrap replicates using MEGA 6.0 software. Labels at branch tips refer to the strain name and GenBank accession number. The black solid dots mark 91 PEDV S sequences detected in this study. Sequences from different genotypes are marked with different color. GI-a, GI-b, S-INDEL like strain, GII-a, and GII-b are labelled with purple, blue, green, orange, and grey, respectively

The length of *S* genes of 91 field strains was 4149–4176 nucleotides (nt), which encoded proteins of 1383–1392 amino acids. A phylogenetic analysis based on the sequenced *S* genes and 24 reference strains indicated that PEDV can be divided into two genotypes (GI and GII) and further classified into five subgroups, including GI-a, GI-b, GI-c, GII-a, and GII-b (Fig. 1). The GI-a clade comprised classical strains, such as CV777, CH/S, and DR13.The classical vaccine strains attenuated from CV777 and DR13 belonged to GI-b subgroup. Notably, 92.3% (84 out of 91) of the strains belonged to genotype GII. CH/HBTS/202010, CH/SCMY/202012, CH/GXLB/202005, and CH/GXLP/202007 were categorized as GII-a and other 80 belonged to GII-b subgroup. Meanwhile, CH/JSXZ/202012, CH/SCYB/202012, CH/SCGA/202103, CH/SCST/202004, CH/HNBR/202101, CH/JXXG/202101, and CH/HNSQ/202102 were located in the GI-c clade and closely related to PEDV S-INDEL strains found in the US (OH851), Korea (KNU-1406-1), and China (ZL29). They distributed in four provinces of China (Table 2).

### Sequence homology analysis

The nucleotide sequences of *S* genes of 91 samples had a homology of 94.2–100%. Four strains in GII-a subgroup shared homology of 97.3–99.7%, while eighty strains in GII-b subgroup shared homology of 96.8–100%. The newly detected seven S-INDEL like strains shared homology of 95.1–95.6% and 94.2–96.8% with non-S-INDEL like strains in subgroups GII-a and GII-b, respectively. The sequence identity among 7 strains in S-INDEL groups was 98.6–99.3% between each other. The strains in GII group shared homology of 93.2–93.8% and 96.7–99.1% with Chinese vaccine strain CV777 (GI-b) and AJ1102 (GII-b), respectively. In addition, the S-INDEL like strains had homology of 94.8–95.2% with Chinese vaccine strains (Table 3).

**Table 3:** Nucleotide acid sequence homology of S genes of 91 PEDV strains and reference strains

Genotype and reference strains		Genotypes		
		GIIa	GII-b	S-INDEL
Percentage Identity (%)	GII-a	97.3–99.7	95.2–97.9	95.1–95.6
	GII-b	95.2–97.9	96.8–100.0	94.2–96.5
	S-INDEL	95.1–95.6	98.6–99.3	98.6–99.3
	CV777	93.2–93.8	92.7–93.7	94.8–95.2
	AJ1102	96.7–97.0	96.9–99.1	94.9–95.1

### Deduced amino acids analysis of PEDV variants in China

**Fig. 2:**
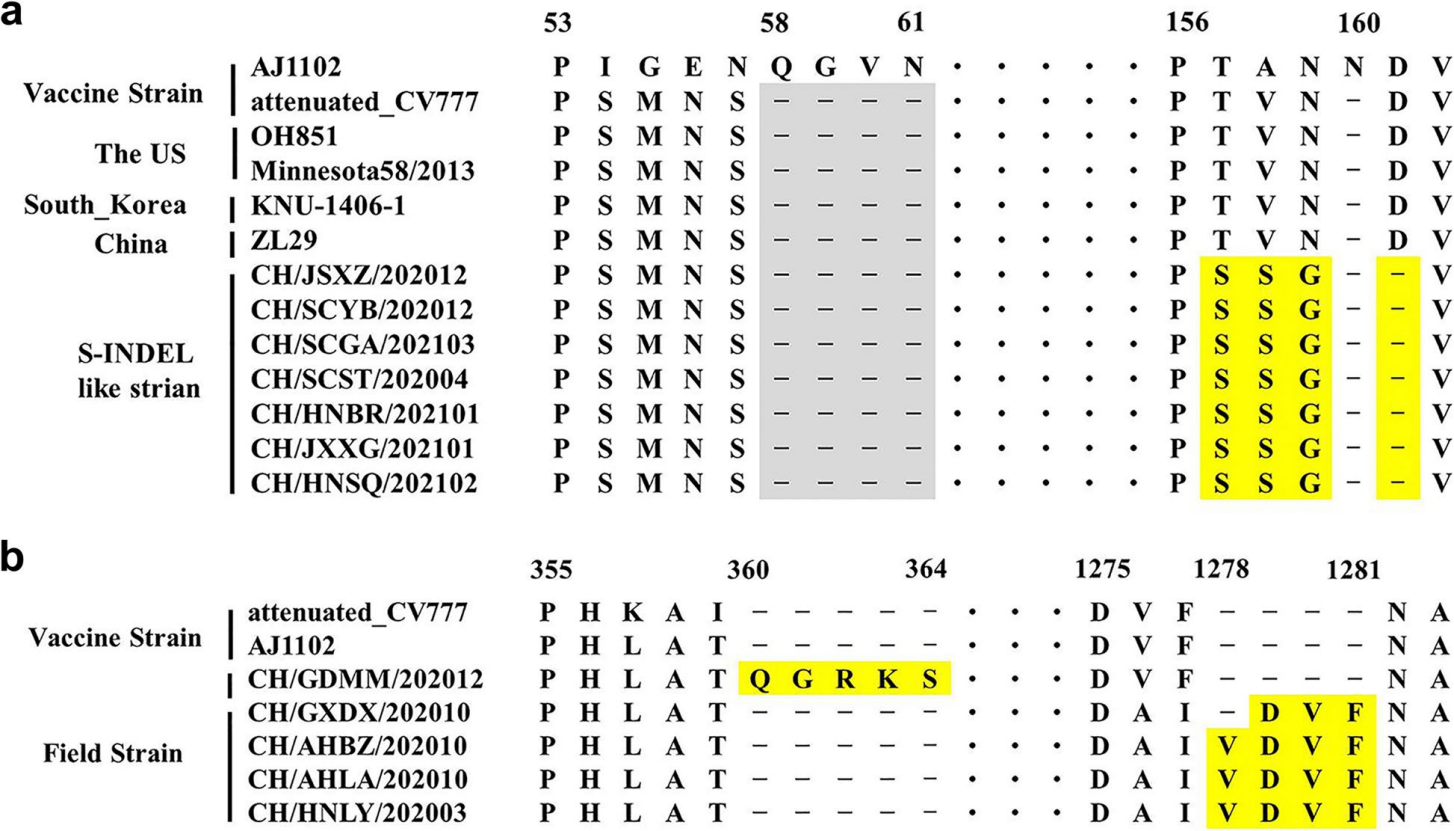
Molecular characterization of the emergent PEDV strains. **a** Alignment of partial S protein sequences of 7 S-INDEL like strains in this study and the prototype S-INDEL strains in the United States (OH851 and Minnesota58), South Korea (KNU-1406-1), and China (ZL29). **b** Locations of unique amino acid (aa) insertions identified in the 91 detected sequences. The symbol “-” indicates an aa deletion. Unique variations of aa are labelled with yellow

In this study, the S-INDEL like strains were identified in seven samples. The deduced amino acid sequences of S proteins in the identified S-INDEL like strains were used to compare with the reference S-INDEL strains. A sequence alignment showed that four amino acid deletions (58QGVN61) were observed in the attenuated CV777 strain (GI-b) and S-INDEL like strains as compared with the virulent strain AJ1102 (GII-b). In addition, three mutations and one deletion of the deduced S protein were found in the identified S-INDEL like strains as compared with the reference S-INDEL strains (Fig. 2a).

A new amino acid insertion relative to the prototype in the S protein were found in five samples. The CH/GDMM/202012 had continuous 5 amino acid insertions (360QGRKS364) in the S1 domain. CH/GXDX/202010, CH/AHBZ/202010, CH/AHLA/202010, and CH/HNLY/202003 had 3 or 4 amino acid insertions (1279DVF1281 or 1278VDVF1281) in the S2 domain (Fig. 2b).

### Recombination analysis of S-INDEL like strains

**Fig. 3:**
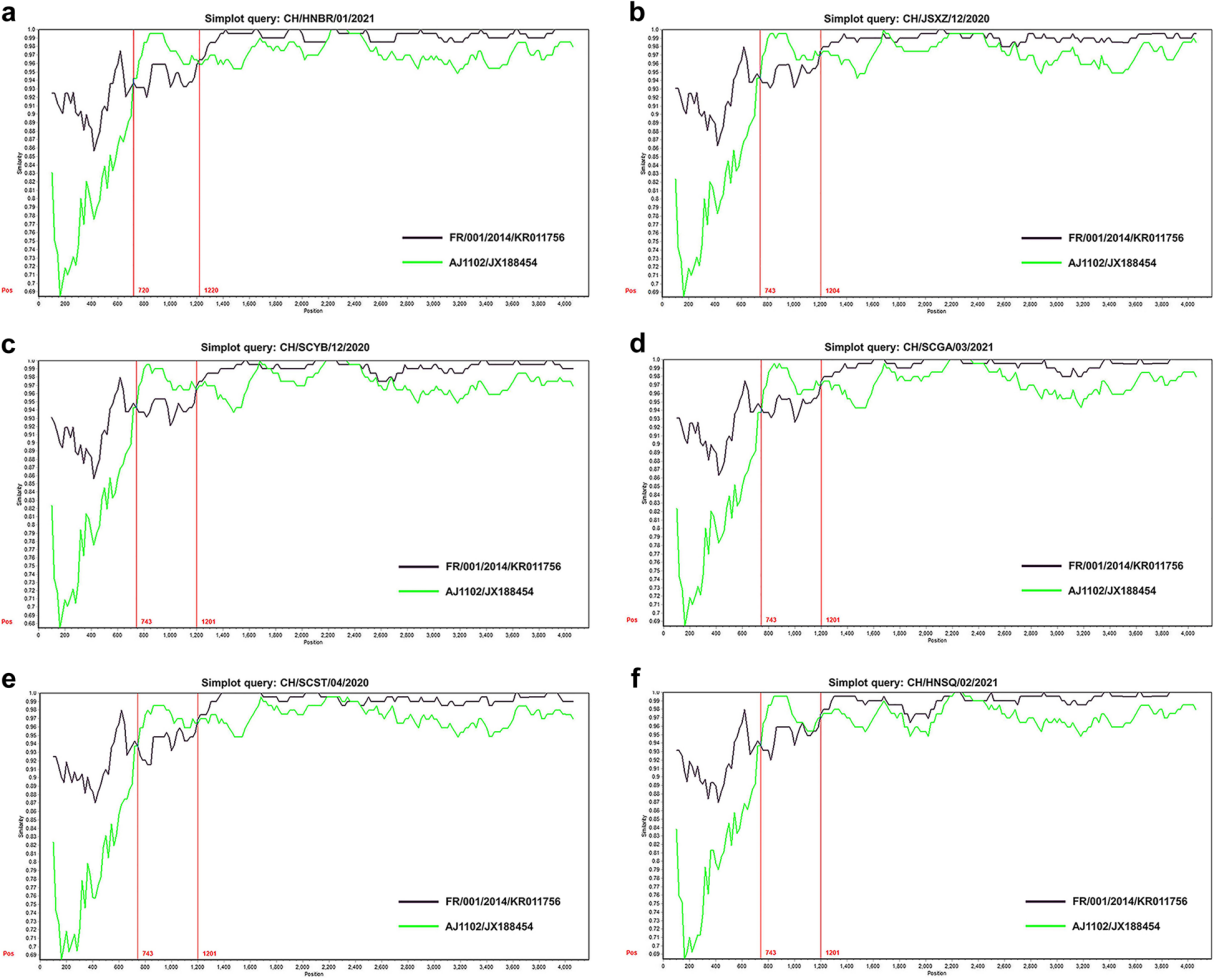
Recombination analysis of six S-INDEL like strains. The recombinant events are identified by using a Simplot analysis. The query sequences from **a** CH/HNBR/01/2021, **b** CH/JSXZ/12/2020, **c** CH/SCYB/12/2020, **d** CH/SCGA/03/2021, **e** CH/SCST/04/2020, and **f** CH/HNSQ/02/2021 were used to compare with the parental sequences FR/001/2014 and AJ1102. The x-axis indicates the *S* gene sequences, and the y-axis represents the similarity value. The regions of recombinant breakpoint are shown within two red lines

A recombination analysis of the 91 strains detected in this study and 178 reference strains were performed by using RDP4 software. The results derived from seven recombination methods indicated that six S-INDEL like strains (CH/HNBR/01/2021, CH/JSXZ/12/2020, CH/SCYB/12/2020, CH/SCGA/202103, CH/SCST/04/2020, and CH/HNSQ/02/2021) were recombinant strains originated from FR/001/2014 strain and virulent strain AJ1102 (Fig. 3). Further similarity comparisons were made using SimPlot among six S-INDEL like strains and their parental strains, the recombination breakpoints were found to be located within the nucleotides 720–1220, 743–1204 or 743–1201 of *S* genes.

### Antigenic epitopes of PEDV S protein in Chinese strains

**Fig. 4:**
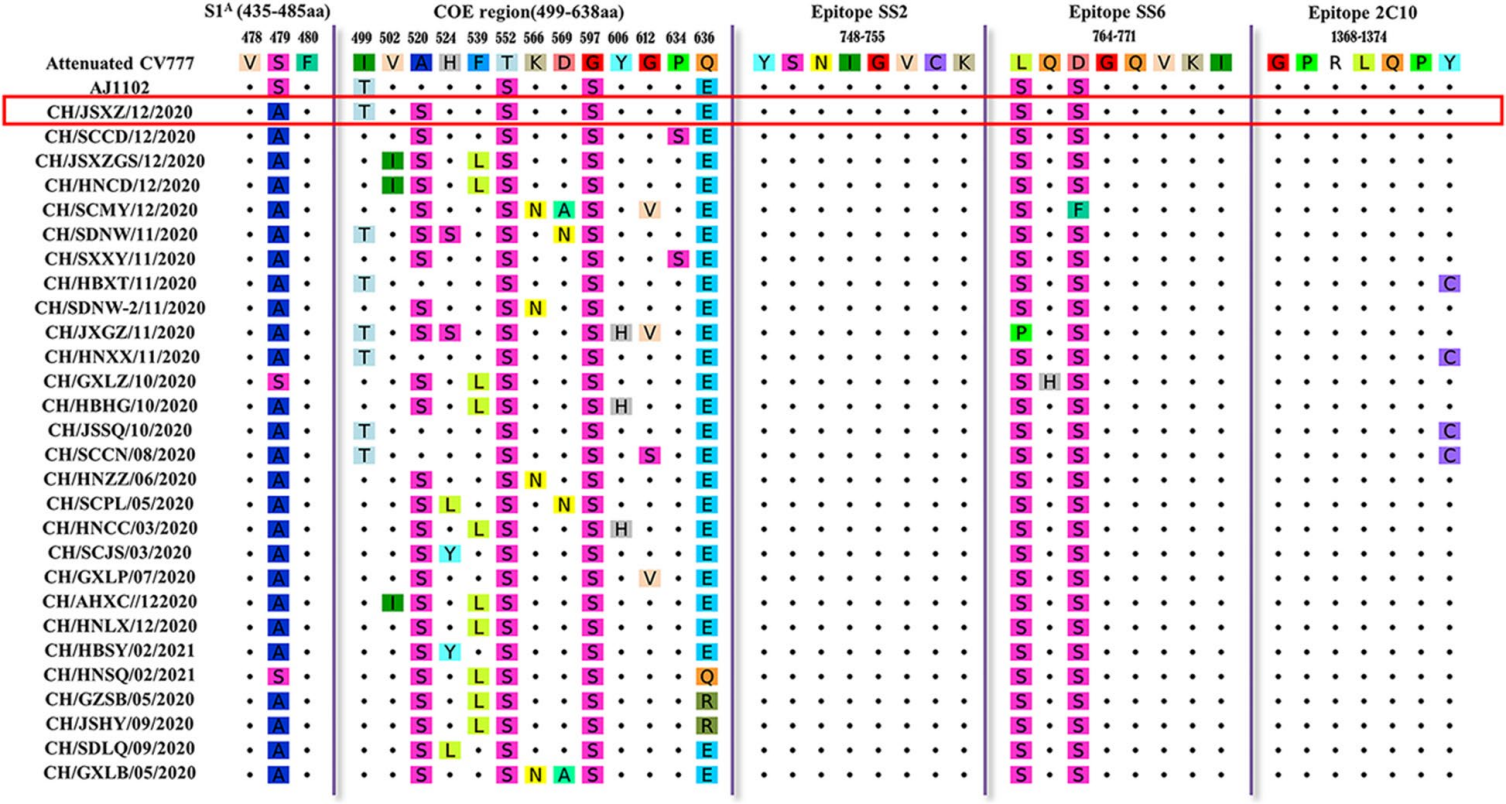
Comparison of the antigen epitopes of S proteins of field strains and the vaccine strains. Dots indicate the amino acids which are identical to those of reference strains. The colored amino acids indicate the mutations in the neutralizing epitopes S1^A^, COE, SS2, SS6, and 2C10. The red box shows the S-INDEL like strain sequence

Five major neutralizing epitopes S1^A^ (435–485aa), COE domain (499–638 aa), SS2 (748–755 aa), SS6 (764–771 aa), 2C10 (1368–1374 aa), and one linear B-cell epitope (SE16, 722–731 aa) have been identified on the surface of S protein. Among these epitopes, SS2, 2C10 and SE16 (data not shown) in all field PEDVs were highly conserved, with only one mutation (1374^Y-C^) in the 2C10 as compared to the two vaccine strains. The aa difference was compared among field strains and vaccine strain CV777 and AJ1102 strains. All of the field strains had two aa substitutions (764^L-S/P^ or 766^D-S/F^). One mutation (765^Q-H^) in the epitope SS6 of CH/GXLZ/202010 was observed. In the S1^A^ epitope, the field strains except for CH/GXLZ/202010 and CH/HNSQ/202102 had one mutation (479^S-A^). In the COE domain, all field strains except for CH/HNSQ/202102 had three substitutions (552^T-S^, 597^G-S^, and 636^Q-E/R^). Meanwhile, a serine substitution (520^A-S^) was observed in all field strains except for CH/HNXX/202011, CH/JSSQ/202010, and CH/SCCN/202008. In addition, multiple mutations on S protein were detected in the COE domain (499^I-T^, 502^V-I^, 524^H-S/L/Y,^ 539^F-L^, 566^K-N^, 569^D-A/N^, 606^Y-H^, 612^G-V/S^, and 634^P-S^) (Fig. 4).

## Discussion

China is the largest country for pig farming in the world, with an annual pig slaughter of approximately 600 million in 2020. The re-emergence of PEDV, especially the variant strains, since 2010 has brought a great threat to Chinese pig industry. PEDV was the major causative agent that led to the diarrheal disease and death in piglets in comparison with other coronaviruses, such as TGEV, PDCoV, and SADS-CoV [23]. Although a variety of commercial vaccines based on classical GI strain CV777 and the variant GII strain AJ1102 have been employed to control the outbreak of PEDV, their efficacies have been always poor. The immunity failure was associated with the constant variation of virus and the insufficient mucosal immunity induced by the vaccines [24]. Therefore, understanding the prevalence and variation characterization of PEDV genome is of importance in preventing PEDV prevalence. In a recent study, 49 complete *S* genes were sequenced, and novel insertions, deletions, and multiple *S* gene recombination events were found in PEDV variant strains [25]. Lei et al. collected 184 specimens from pig farms in China in 2017–2018, and detected an average PEDV-positive rate of 38.04% [26]. Zhang et al. made a survey of molecular characteristics of PEDV in Henan province, China in 2015–2019 and found PEDV existed widely in both PEDV-vaccine immunized (25.00%) and non-immunized swine herds (62.29%). Sixteen of the sequenced PEDV Henan strains were located in the GII clade [27]. However, there is no nationwide epidemiological survey of PEDV in the past 2 years. Furthermore, African swine fever (ASF) outbreaks were declared in China since 2018 and quickly swept through the whole country [28]. Management and prevention measures of epidemic diseases have been altered in pig farms. It is still unknown about if the prevalence characterization of PEDV has changed under the background of ASF. In this study, 115 PEDV positive samples were collected in 17 provinces of China, including large swine-raising provinces, such as Sichuan, Henan, Hunan, and Shandong province. Our data showed that the positive rate of PEDV was from 10.8–25.1% from March 2020 to April 2021, thus indicating that PEDV was still one of the major pathogenic agents in Chinese swine herds. Our data also showed that the positive rate of PEDV was the highest in October and December. Thus, we should pay more attention to the prevention of PEDV when the weather turns cold, such as the turn of autumn and winter and the winter.

The full-length nucleotide sequences of *S* genes of 91 field strains were successfully sequenced. Based on the phylogenetic analysis for the *S* gene, all of the PEDV strains could be classified into two main genotypes and five subgroups, including GI-a (classical strain), GI-b (attenuated classical strain), GI-c (S-INDEL strain), GII-a, and GII-b (variant strain). All strains detected in this study shared 94.2–100% homology between each other. A majority of them belonged to GII genotype (84 out of 91) which represented the pandemic strains of PEDV in recent years. GII genotype strains shared 93.2–93.8% and 96.7–99.1% homology with attenuated Chinese vaccine strains CV777 and AJ1102, respectively. Vaccines based on CV777 strain was widely used and contributed to a good control of PEDV in China before 2010 [29]. However, the variant strains that were clustered into different subgroup (GII) shared lower homology with CV777 (GI). The different genotypes between CV777 and PEDV epidemic strains may lead to an incomplete protection provided by the vaccines in China since 2010.

The detected strains CH/JSXZ/202012, CH/SCYB/202012, CH/SCGA/202103, CH/SCST/202004, CH/HNBR/202101, CH/JXXG/202101, and CH/HNSQ/202102 were more related to S-INDEL strains. They were obtained from four provinces of China and classified into GI-c subgroup, thus suggesting that S-INDEL like strains have spread and circulated in China. Seven of S-INDEL like strains shared a high homology of 98.6–99.3% between each other. Although the presence of S-INDEL strains of PEDV has been described to induce less severe symptoms and low fatality rate as compared with non-INDEL strains [30, 31], the pathogenicity of this subgroup is still controversial. Severe diarrhea and vomiting, along with high mortality rates were associated with the S-INDEL PEDV strains in some European countries [32]. Therefore, the pathogenicity and infection rate of S-INDEL like strains in China should be constantly monitored.

**Fig. 5:**
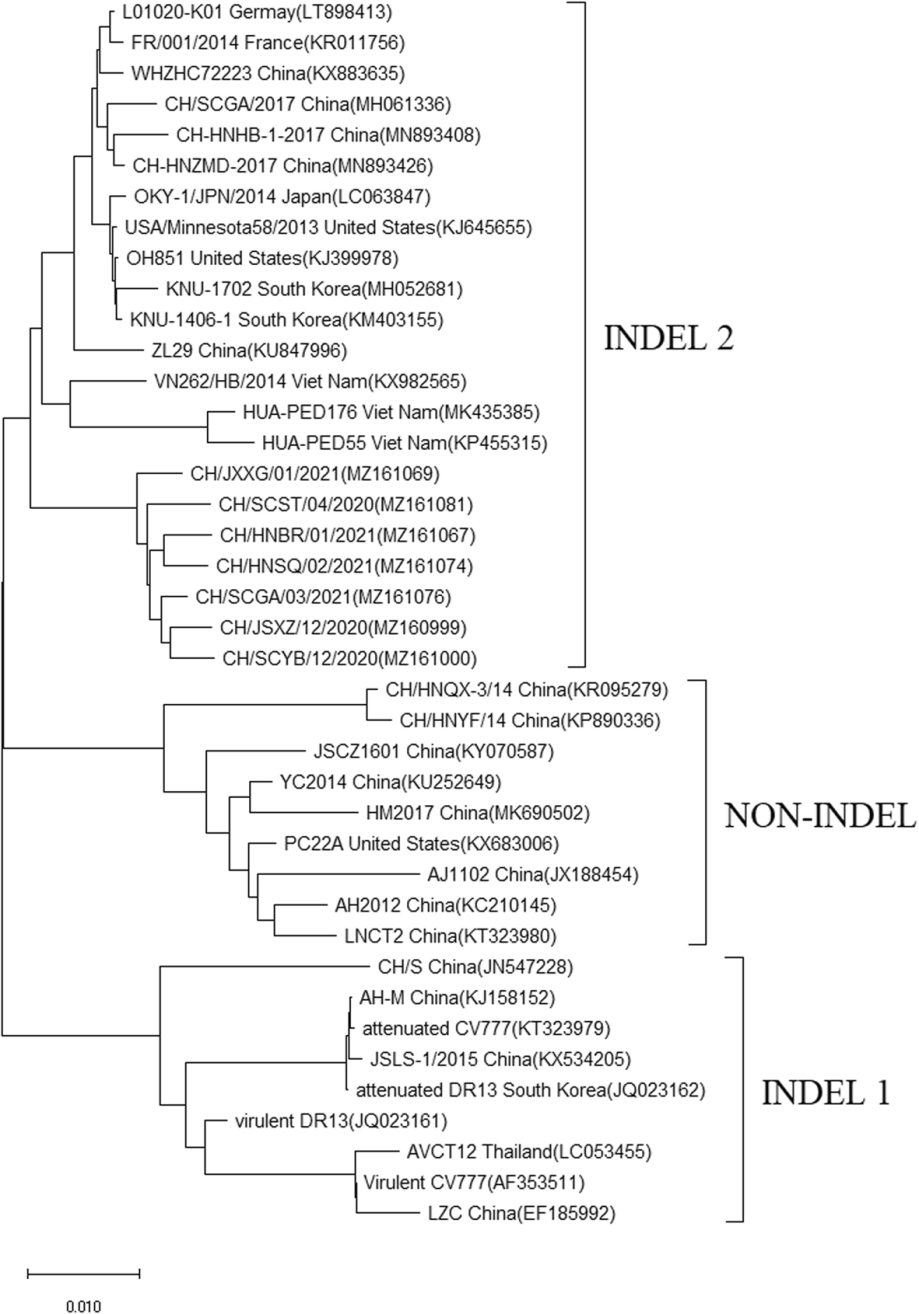
Phylogenetic analysis based on the *S* genes of S-INDEL like strains and the reference S-INDEL strains around the world. The evolutionary tree was constructed by the neighbor-joining method using MEGA6 software. Bootstrap values are indicated for each node, based on 1000 replicates. The positions of seven S-INDEL like strain are annotated by solid black circles

To date, S-INDEL strains have been detected widely in the world, including America, Asia, and Europe. The S-INDEL strains could be separated into two distant clades (INDEL 1 and INDEL 2) based on the characteristics of the S-INDEL genotype [33]. INDEL 2 contained prototype American (OH851) and European strains (FR/001/2014), as well as the first identified Chinese S-INDEL strain ZL29. Some other Asia S-INDEL strains belonged to the INDEL 2 clade. A further phylogenetic analysis using the seven S-INDEL like strains and reference S-INDEL strains retrieved from the NCBI nucleotide database showed that all of the detected S-INDEL like strains were categorized into INDEL 2. However, they were clearly clustered within a new cluster in INDEL 2 clade (Fig. 5). Although the identified seven S-INDEL like strains had the common four aa deletion (58QGVN61) which was in line with the reference S-INDEL strains, they had additional novel three amino acid mutations and one deletion located at the positions 156–160 aa. As previously described, INDEL 2 clade might originate from virulent strains DR13, Italy/7239/2009 or other field NON-INDEL strains, whereas INDEL 1 showed a common ancestor, including CV777 or other PEDV strains detected in China before 2010 [24, 34]. According to our results, the novel S-INDEL like strains detected in this study were originated from FR/001/2014 in INDEL 2 clade and virulent strain AJ1102. This indicated that natural recombinant events might exist among variant strains and vaccine strains in China. Therefore, the use of live vaccine strains should be carefully considered especially in PEDV positive pig farms.

Spike protein is the most variable protein of PEDV. The variation of S protein is considered to be responsible for the changes of viral antigenicity, determination of the genetic diversity for PEDV and affected the viral virulence [35, 36]. To evaluate whether the antigenicity of PEDV field strains has changed as compared with vaccine strains used in China, the neutralizing antigenic epitopes on S protein were analyzed. Several single nucleotide polymorphisms (SNPs) were observed on the S1^A^，SS6，and 2C10 epitopes, and COE domain. Five main serine substitution (520^A-S^, 552^T-S^, 597^G-S^, 764^L-S^, and 766^D-S^) and one Glutamate substitution (636^Q-E^) were found in the field strains as compared with the vaccine strain CV777. One common substitution in S1^A^ (479^S-A^) and multiple mutations (499^I-T^, 502^V-I^, 524^H-S/L/Y^, 539^F-L^, 566^K-N^, 569^D-A/N^, 606^Y-H^, 612^G-V/S^, and 634^P-S^) in individual sequences were detected in the field stains as compared with the virulent strain AJ1102. These mutations might involve in the immune escape and the antigenicity of virus, resulting in a less effective immune protection provided by the commercial vaccines CV777 and AJ1102. Notably, five PEDV samples with new insertion in the S protein sequences were detected. CH/GDMM/202012 had a continuous 5 aa insertion (360QGRKS364) located in S1 domain while the other four (CH/GXDX/202010, CH/AHBZ/202010, CH/AHLA/202010, and CH/HNLY/202003) had three or four amino acid insertions (1279DVF1281 or 1278VDVF1281) located in S2 domain. To the best of our knowledge, this is the first report for these novel S-insertion variants. The S-INDEL-variants were proved to have decreased virulence in host, whereas one strain with a novel four-amino-acid insertion in the COE domain was highly pathogenic to the neonatal pigs [37]. Although we did not obtain the isolated novel S-insertion strains, it could be predicted that the conformational structure of S protein might have changed, probably resulting in a change for the pathogenicity of these strains.

## Conclusions

Collectively, this study described the nationwide investigation of the PEDV prevalence in China in recent years. The major causative virus strains were GII genotype variants with the ratio of 92.3%. Notably, seven S-INDEL like strains were detected in four provinces in China. Alignment of S deduced aa sequences revealed novel mutations and deletion compared with prototype S-INDEL strain. Based on recombination analyses, the novel S-INDEL like strains were originated from FR/001/2014 in INDEL 2 clade and virulent strain AJ1102. Moreover, variant PEDV strains with novel insertions (360QGRKS364 and 1278VDVF1281) in S protein sequences were detected and needed to be addressed on the specific function of insertions in the future work. In addition, we also identified multiple mutations in the aa sequences of S proteins of the variant strains compared to those of the vaccine strains. These PEDV mutants derived from genetic mutations, deletions, insertions, and recombination of the *S* genes might be the major cause of antigenic drift and immune failure. The molecular characterization of S protein should be investigated continuously and would work in the control and prevention of PED in China.

## Materials and methods

### Sample collection

A total of 115 field PEDV positive samples (intestine, intestinal content, feces, and anal swab) were collected from various pig farms located in 17 provinces of China from March 2020 to March 2021. Animal samples were collected from the farms of New Hope Liuhe Co., Ltd. and Jiangxi Zhengbang Technology Co., Ltd., respectively. We have acquired a consent from the farm owners. All samples were stored at − 80 °C before RNA extraction.

### PCR detection of PEDV

The samples were diluted with phosphate-buffered saline and centrifuged at 8,000×g for 10 min at 4 °C and the supernatants were transferred into a 1.5 mL RNase-free tube. Viral RNA was extracted using the TIANamp Virus DNA/RNA Kit (TIANGEN) according to the manufacturer’s instructions. Reverse transcription was carried out using PrimeScript™ IV 1st strand cDNA Synthesis Mix (TaKara). The full-length *S* gene of PEDV was amplified using EmeraldAmp® PCR Master Mix (TaKara). The S1 was amplified by two pairs of primers (S1-1U, 5′- ATCGTCAGAGGCATTTTTAA-3′; S1-1L, 5′-ATCCATCACCATTAAACGAA-3′; S1-2U, 5′-ATGTTGTGTTAGGCTTGTTG-3′; S1-2L, 5′-CACTAACAGGCGTGTTGTAA-3′), and the S2 was amplified by three primers (S2-U, 5′-CTGATTCTGGACAGTTGTTA-3′; S2-1L, 5′-TTGGACAGCATCCAAAGACA-3′; S2-2L, 5′-CTTCGAGACATCTTTGACAA-3′) [38]. PCR products were purified and recovered using AxyPrep™ DNA Gel Extraction Kit (Axygen), and then subjected to sequencing by Sangon Biotech company (Shanghai, China).

### Genetic and phylogenetic analysis

The 91 complete *S* gene sequences of PEDV obtained in this study have been uploaded to GenBank with accession numbers from MZ160999 to MZ161089. Twenty-four reference sequences retrieved from the NCBI nucleotide database and the 91 identified sequences were used for the phylogenetic analysis. Multiple nucleotide sequence alignments were performed with Clustal W algorithm using MEGA 6.0 software. A phylogenetic tree based on *S* gene sequence was constructed by the neighbor-joining method using MEGA 6.0 software, with bootstrap values calculated for each node from 1000 replicates [39, 40].

### Deduced amino acid analysis of PEDV variants

To elucidate the genetic characteristics of S-INDEL like strains detected in this study, partial *S* gene sequences were aligned with reference S-INDEL strains from US (OH851), South Korea (KNU-1406-1), and China (ZL29) using MEGA 6.0 software. The novel mutation, insertion, and deletion in S proteins were also detected in this study.

### Recombination analysis

In order to investigate the recombination events occurred in the detected variant strains, especially the S-INDEL, all of the detected strains and 178 reference strains obtained from Genbank were analyzed using program RDP4 [41]. Potential recombinant strains, parental strains, and possible recombination breakpoints were identified by a series of methods including RDP, GeneConv, SiScan, 3Seq, BootScan, MaxChi, and Chimaera. Recombination events were examined by at least three of the methods mentioned above with a cutoff of *p* < 0.05. Based on the fragment size of genome that contributed to the recombinants, the original sequences were defined as major parents (contributing the larger fragment) and the minor parents (contributing the small fragment). The recombinant strain was further analyzed by Simplot v3.5.1 software [42].

### Analysis of the antigenic epitopes on the S proteins

In order to study the antigenic variation of PEDV strains, all of the ninety-one S protein sequences were used for a sequence alignment. Twenty-eight representative sequences were selected and compared with Chinese PEDV vaccine strain (attenuated CV777 and AJ1102). A focus was on the neutralizing epitope regions including S1^A^, COE domain, SS2, SS6, 2C10, and the linear B-cell epitope SE16 [43, 44].

## Additional Files

**Additional file 1: Table S1.** Information of PEDV strains obtained in this study. The information including strain name, collection date, collection region, *S* gene length, virus isolation and accession number.

## Data Availability

The nucleotide sequence data obtained in this study are available in GenBank, and their accession numbers are MZ160999-MZ161089. The nucleic acid sequences referenced in this paper were obtained from National Center for Biotechnology Information (https://www.ncbi.nlm.nih.gov/nuccore).

## Abbreviations

PEDV: Porcine epidemic diarrhea virus
S: Spike
CoVs: Coronaviruses
TGEV: Transmissible gastroenteritis virus
PDCoV: Porcine deltacoronavirus
SADS-CoV: Swine acute diarrhea syndrome coronavirus
ORF: Open reading frame
E: Envelope
M: Membrane
N: Nucleoprotein
UTR: Untranslated region
aa: Amino acids
pAPN: Porcine aminopeptidase N
nt: Nucleotides
ASF: African swine fever
SNPs: Single nucleotide polymorphisms
