# Supplementary material for: Molecular characterization and phylogenetic analysis of porcine epidemic diarrhea virus strains circulating in China from 2020 to 2021
Source: BMC Vet Res. 2022 Nov 8;18:392. doi: 10.1186/s12917-022-03481-4 (PMC9643976; doi:10.1186/s12917-022-03481-4)
Supplement: Supplementary file 1 — Additional file 1: Table S1. Information of PEDV strains obtained in this study. The information including strain name, collection date, collection region, S gene length, virus isolation and accession number. [file 12917_2022_3481_MOESM1_ESM.docx]

Molecular characterization and phylogenetic analysis of porcine epidemic diarrhea virus strains circulating in China from 2020 to 2021

Running title: Molecular epidemiology of PEDV

Hong Zhuang^1†^, Leilei Sun^2†^, Xiaobo Wang^2,3†^, Min Xiao^4†^, Long Zeng^4^, Haoran Wang^1^, Hongfu Yang^4^, Feng Lin^4^, Chuang Wang^4*^, Liting Qin^2,3*^, Chengbao Wang^1*^

^1^College of Veterinary Medicine, Northwest A&F University, Yangling, 712100, China

^2^Shandong New Hope Liuhe Group Co., Ltd., Qingdao, 266000, China

^3^Qingdao Jiazhi Biotechnology Co., Ltd., Qingdao, 266000, China

^4^Zhengbang Group Co., Ltd., Nanchang, 330000, China

*** Correspondence:**

Chuang Wang

Zhengbang Group Co., Ltd., Nanchang, China

E-mail addresses: [wangchuang@zhengbang.com](mailto:wangchuang@zhengbang.com)

Liting Qin

Shandong New Hope Liuhe Group Co., Ltd., Qingdao, China

Qingdao Jiazhi Biotechnology Co., Ltd., Qingdao, China

E-mail addresses: [qinlt@newhope.cn](mailto:qinlt@newhope.cn)

Chengbao Wang

College of Veterinary Medicine, Northwest A&F University, Yangling, China

Phone: +86-18049569130; Fax: +86-29-87091032

E-mail addresses: wangchengbao@nwafu.edu.cn

^†^ These authors have contributed equally to this work and share first authorship.

**Supplementary Table** **1** Information of PEDV strains obtained in this study.

| No. | Name | Collection Date | Collection Region | abbreviation of sampling provinces | S gene length (bp) | Virus isolation | Genotype | Accession number |
| --- | --- | --- | --- | --- | --- | --- | --- | --- |
| 1 | CH/JSXZ/12/2020 | 2020.12 | Jiangsu/China | JS | 4149 | No | S INDEL like strain | MZ160999 |
| 2 | CH/SCYB/12/2020 | 2020.12 | SiChuan/China | SC | 4149 | No | S INDEL like strain | MZ161000 |
| 3 | CH/GDHG/12/2020 | 2020.12 | Guangdong/China | GD | 4158 | No | GII-b | MZ161001 |
| 4 | CH/GDSG/12/2020 | 2020.12 | Guangdong/China | GD | 4161 | No | GII-b | MZ161002 |
| 5 | CH/SCYBZH/12/2020 | 2020.12 | Sichuan/China | SC | 4161 | No | GII-b | MZ161003 |
| 6 | CH/GXGG/12/2020 | 2020.12 | Guangxi/China | GX | 4161 | No | GII-b | MZ161004 |
| 7 | CH/JSHA/12/2020 | 2020.12 | Jiangsu/China | JS | 4161 | No | GII-b | MZ161005 |
| 8 | CH/JSSZ/12/2020 | 2020.12 | Jiangsu/China | JS | 4161 | No | GII-b | MZ161006 |
| 9 | CH/HBSY/12/2020 | 2020.12 | Hubei/China | HB | 4161 | No | GII-b | MZ161007 |
| 10 | CH/GDMM/12/2020 | 2020.12 | Guangdong/China | GD | 4176 | No | GII-b | MZ161008 |
| 11 | CH/SCCD/12/2020 | 2020.12 | Sichuan/China | SC | 4158 | No | GII-b | MZ161009 |
| 12 | CH/JSXZGS/12/2020 | 2020.12 | Jiangsu/China | JS | 4161 | No | GII-b | MZ161010 |
| 13 | CH/HNCD/12/2020 | 2020.12 | Hunan/China | HN | 4161 | No | GII-b | MZ161011 |
| 14 | CH/SCMY/12/2020 | 2020.12 | Sichuan/China | SC | 4161 | No | GII-a | MZ161012 |
| 15 | CH/AHBB/12/2020 | 2020.12 | Anhui/China | AH | 4161 | No | GII-b | MZ161013 |
| 16 | CH/GDYD/12/2020 | 2020.12 | Guangdong/China | GD | 4158 | No | GII-b | MZ161014 |
| 17 | CH/SDNW/11/2020 | 2020.11 | Shandong/China | SD | 4158 | No | GII-b | MZ161015 |
| 18 | CH/JXXG/11/2020 | 2020.11 | Jiangxi/China | JX | 4161 | No | GII-b | MZ161016 |
| 19 | CH/SXXY/11/2020 | 2020.11 | Shaanxi/China | SN | 4158 | No | GII-b | MZ161017 |
| 20 | CH/HBXT/11/2020 | 2020.11 | Hebei/China | HE | 4158 | No | GII-b | MZ161018 |
| 21 | CH/YNDL/11/2020 | 2020.11 | Yunnan/China | YN | 4158 | No | GII-b | MZ161019 |
| 22 | CH/SDNW-2/11/2020 | 2020.11 | Shandong/China | SD | 4161 | No | GII-b | MZ161020 |
| 23 | CH/JXGZ/11/2020 | 2020.11 | Jiangxi/China | JX | 4158 | No | GII-b | MZ161021 |
| 24 | CH/HNXX/11/2020 | 2020.11 | Hunan/China | HN | 4158 | No | GII-b | MZ161022 |
| 25 | CH/GXDX/10/2020 | 2020.10 | Guangxi/China | GX | 4167 | No | GII-b | MZ161023 |
| 26 | CH/HBBX/10/2020 | 2020.10 | Hebei/China | HE | 4164 | No | GII-b | MZ161024 |
| 27 | CH/HBSZ/10/2020 | 2020.10 | Hubei/China | HB | 4158 | No | GII-b | MZ161025 |
| 28 | CH/HBMZ/10/2020 | 2020.10 | Hebei/China | HE | 4158 | No | GII-b | MZ161026 |
| 29 | CH/GXLZ/10/2020 | 2020.10 | Guangxi/China | GX | 4161 | No | GII-b | MZ161027 |
| 30 | CH/HBHG/10/2020 | 2020.10 | Hubei/China | HB | 4161 | No | GII-b | MZ161028 |
| 31 | CH/HBHS/10/2020 | 2020.10 | Hebei/China | HE | 4158 | No | GII-b | MZ161029 |
| 32 | CH/AHBZ/10/2020 | 2020.10 | Anhui/China | AH | 4170 | No | GII-b | MZ161030 |
| 33 | CH/HBTS/10/2020 | 2020.10 | Hubei/China | HB | 4161 | No | GII-a | MZ161031 |
| 34 | CH/JSSQ/10/2020 | 2020.10 | Jiangsu/China | JS | 4158 | No | GII-b | MZ161032 |
| 35 | CH/JSJH/10/2020 | 2020.10 | Jiangsu/China | JS | 4158 | No | GII-b | MZ161033 |
| 36 | CH/SCPZ/10/2020 | 2020.10 | Sichuan/China | SC | 4161 | No | GII-b | MZ161034 |
| 37 | CH/AHLA/10/2020 | 2020.10 | Anhui/China | AH | 4170 | No | GII-b | MZ161035 |
| 38 | CH/SCLZ/09/2020 | 2020.09 | Sichuan/China | SC | 4161 | No | GII-b | MZ161036 |
| 39 | CH/SCCN/08/2020 | 2020.08 | Sichuan/China | SC | 4158 | No | GII-b | MZ161037 |
| 40 | CH/JXHY/09/2020 | 2020.09 | Jiangxi/China | JX | 4158 | No | GII-b | MZ161038 |
| 41 | CH/LNFS/09/2020 | 2020.09 | Liaoning/China | LN | 4161 | No | GII-b | MZ161039 |
| 42 | CH/GXLA/07/2020 | 2020.07 | Guangxi/China | GX | 4161 | No | GII-b | MZ161040 |
| 43 | CH/JXPY/07/2020 | 2020.07 | Jiangxi/China | JX | 4152 | No | GII-b | MZ161041 |
| 44 | CH/SDZH/07/2020 | 2020.07 | Shandong/China | SD | 4161 | No | GII-b | MZ161042 |
| 45 | CH/HBCZ/07/2020 | 2020.07 | Hebei/China | HE | 4161 | No | GII-b | MZ161043 |
| 46 | CH/AHMJ/07/2020 | 2020.07 | Anhui/China | AH | 4161 | No | GII-b | MZ161044 |
| 47 | CH/GDCY/06/2020 | 2020.06 | Guangdong/China | GD | 4161 | No | GII-b | MZ161045 |
| 48 | CH/SCMY/06/2020 | 2020.06 | Sichuan/China | SC | 4158 | No | GII-b | MZ161046 |
| 49 | CH/HNZZ/06/2020 | 2020.06 | Henan/China | HA | 4161 | No | GII-b | MZ161047 |
| 50 | CH/LNPJ/05/2020 | 2020.05 | Liaoning/China | LN | 4161 | No | GII-b | MZ161048 |
| 51 | CH/SCPL/05/2020 | 2020.05 | Sichuan/China | SC | 4161 | No | GII-b | MZ161049 |
| 52 | CH/GXNN/04/2020 | 2020.04 | Guangxi/China | GX | 4161 | No | GII-b | MZ161050 |
| 53 | CH/GXLB/04/2020 | 2020.04 | Guangxi/China | GX | 4161 | No | GII-b | MZ161051 |
| 54 | CH/JXJJ/04/2020 | 2020.04 | Jiangxi/China | JX | 4161 | Yes | GII-b | MZ161052 |
| 55 | CH/GDCY/04/2020 | 2020.04 | Guangdong/China | GD | 4161 | No | GII-b | MZ161053 |
| 56 | CH/FJDH/04/2020 | 2020.04 | Fujian/China | FJ | 4164 | No | GII-b | MZ161054 |
| 57 | CH/JXGZ/04/2020 | 2020.04 | Jiangxi/China | JX | 4161 | No | GII-b | MZ161055 |
| 58 | CH/HNCC/03/2020 | 2020.03 | Hunan/China | HN | 4161 | No | GII-b | MZ161056 |
| 59 | CH/HBZL/03/2020 | 2020.03 | Hubei/China | HB | 4161 | No | GII-b | MZ161057 |
| 60 | CH/SCJS/03/2020 | 2020.03 | Sichuan/China | SC | 4161 | No | GII-b | MZ161058 |
| 61 | CH/HNLY/03/2020 | 2020.03 | Hunan/China | HN | 4170 | No | GII-b | MZ161059 |
| 62 | CH/GXLP/07/2020 | 2020.07 | Guangxi/China | GX | 4161 | Yes | GII-a | MZ161060 |
| 63 | CH/HBZJC/11/2020 | 2020.11 | Hubei/China | HB | 4158 | Yes | GII-b | MZ161061 |
| 64 | CH/AHXC/12/2020 | 2020.12 | Anhui/China | AH | 4161 | No | GII-b | MZ161062 |
| 65 | CH/GZZY/12/2020 | 2020.12 | Guizhou/China | GZ | 4158 | No | GII-b | MZ161063 |
| 66 | CH/JXPY/12/2020 | 2020.12 | Jiangxi/China | JX | 4161 | No | GII-b | MZ161064 |
| 67 | CH/HNLX/12/2020 | 2020.12 | Hunan/China | HN | 4161 | No | GII-b | MZ161065 |
| 68 | CH/HBXY/12/2020 | 2020.12 | Hubei/China | HB | 4158 | No | GII-b | MZ161066 |
| 69 | CH/HNBR/01/2021 | 2021.01 | Henan/China | HA | 4149 | No | S INDEL like strain | MZ161067 |
| 70 | CH/JSXZ/01/2021 | 2021.01 | Jiangsu/China | JS | 4161 | No | GII-b | MZ161068 |
| 71 | CH/JXXG/01/2021 | 2021.01 | Jiangxi/China | JX | 4149 | No | S INDEL like strain | MZ161069 |
| 72 | CH/JXXG-2/01/2021 | 2021.01 | Jiangxi/China | JX | 4161 | No | GII-b | MZ161070 |
| 73 | CH/HBHA/01/2021 | 2021.01 | Hubei/China | HB | 4161 | No | GII-b | MZ161071 |
| 74 | CH/JSLYG/02/2021 | 2021.02 | Jiangsu/China | JS | 4161 | No | GII-b | MZ161072 |
| 75 | CH/HBSY/02/2021 | 2021.02 | Hubei/China | HB | 4161 | No | GII-b | MZ161073 |
| 76 | CH/HNSQ/02/2021 | 2021.02 | Henan/China | HA | 4149 | No | S INDEL like strain | MZ161074 |
| 77 | CH/GDYJ/03/2021 | 2021.03 | Guangdong/China | GD | 4161 | No | GII-b | MZ161075 |
| 78 | CH/SCGA/03/2021 | 2021.03 | Sichuan/China | SC | 4149 | No | S INDEL like strain | MZ161076 |
| 79 | CH/SDLY/12/2020 | 2020.12 | Shandong/China | SD | 4161 | No | GII-b | MZ161077 |
| 80 | CH/SCYT/05/2020 | 2020.05 | Sichuan/China | SC | 4158 | No | GII-b | MZ161078 |
| 81 | CH/SCDY/05/2020 | 2020.05 | Sichuan/China | SC | 4161 | No | GII-b | MZ161079 |
| 82 | CH/GZSB/05/2020 | 2020.05 | Guizhou/China | GZ | 4161 | No | GII-b | MZ161080 |
| 83 | CH/SCST/04/2020 | 2020.04 | Sichuan/China | SC | 4149 | No | S INDEL like strain | MZ161081 |
| 84 | CH/JSHY/09/2020 | 2020.09 | Jiangsu/China | JS | 4161 | No | GII-b | MZ161082 |
| 85 | CH/HBTS/09/2020 | 2020.09 | Hebei/China | HE | 4152 | No | GII-b | MZ161083 |
| 86 | CH/JSXZLD/10/2020 | 2020.10 | Jiangsu/China | JS | 4161 | No | GII-b | MZ161084 |
| 87 | CH/SDLQ/09/2020 | 2020.09 | Shandong/China | SD | 4158 | No | GII-b | MZ161085 |
| 88 | CH/GXLB/05/2020 | 2020.05 | Guangxi/China | GX | 4161 | Yes | GII-a | MZ161086 |
| 89 | CH/NMGTL/04/2020 | 2020.04 | Inner Mongolia/China | NM | 4158 | No | GII-b | MZ161087 |
| 90 | CH/SCLZ/12/2020 | 2020.12 | Sichuan/China | SC | 4161 | No | GII-b | MZ161088 |
| 91 | CH/SDJN/07/2020 | 2020.07 | Shandong/China | SD | 4158 | No | GII-b | MZ161089 |
